# Inhibitory effect of valproic acid on bladder cancer in combination with chemotherapeutic agents *in vitro* and *in vivo*

**DOI:** 10.3892/ol.2013.1565

**Published:** 2013-09-06

**Authors:** DEGUI WANG, YUHONG JING, SIWEI OUYANG, BEI LIU, TIANYUAN ZHU, HAITAO NIU, YINGXIA TIAN

**Affiliations:** 1 Department of Anatomy, School of Basic Medical Sciences, Lanzhou University, Lanzhou, Gansu, P.R. China; 2 Gansu Medical Research Institute, Lanzhou, Gansu, P.R. China; 3 Department of Urology, The Affiliated Hospital of Medical College Qingdao University, Qingdao, Shandong, P.R. China

**Keywords:** valproic acid, apoptosis, combination, bladder cancer, therapy

## Abstract

Histone deacetylase inhibitors (HDACIs) are a promising class of drugs that act as antiproliferative agents by promoting differentiation and inducing apoptosis. Valproic acid (VPA) is an HDACI that has been widely used as an anti-convulsant and shows promise as a chemotherapeutic drug for a number of tumor cells. The present study aimed to investigate the inhibitory effect of VPA on the viability of bladder cancer cells and its synergistic effect with chemotherapeutic agents *in vitro* and *in vivo*. The cell viability of human bladder cancer cell lines following treatment with VPA and/or VPA in combination with mitomycin C, cisplatin (DDP) and adriamycin were determined using a 3-(4,5-dimethylthiazol-2-yl)-2,5-diphenyltetrazolium bromide assay. Hoechst staining was used to observe the morphology of the apoptotic cells. Survivin protein and acetylated histone H3 levels were quantified using western blot analysis. The *in vivo* tumor growth inhibition of VPA was determined in rats with N-methyl-N-nitrosourea-induced bladder cancer. VPA significantly inhibited the growth of the bladder cancer cells in a concentration- and time-dependent manner. Furthermore, improved results were achieved for tumor inhibition when VPA was combined with chemotherapeutic agents *in vitro* and *in vivo*. Survivin expression decreased and acetylated histone H3 expression increased in the bladder cancer cells following the treatment with VPA. Intravesical injections of VPA were able to inhibit tumor progression when combined with DDP. In conclusion, VPA acts as an HDACI that has a direct anticancer effect and markedly enhances the action of several chemotherapy agents. VPA may sensitize bladder cancer to anticancer drugs by downregulating survivin expression.

## Introduction

Bladder cancer is a commonly occurring cancer. Existing local therapies for transitional cell carcinoma (TCC) of the bladder include local resection for non-muscle-invasive disease and cystectomy for muscle-invasive disease. These strategies are effective but far from satisfactory. Between 50 and 70% of patients that are treated for superficial diseases develop recurrences and 20% progress to more aggressive disease (1). Furthermore, chemotherapy and radiotherapy produce disappointing results, which means that new therapeutic approaches are required. An increasing body of evidence concerning the significance of the epigenetic changes in the onset and progression of cancer has raised interest in the manipulation of transcription as a mode of cancer therapy (2). Altering gene expression through chromatin modification now appears to be a viable target. Consistent with this, histone deacetylase inhibitors (HDACIs) have emerged as promising anticancer drugs (2).

The acetylation of core nucleosomal histones is regulated by the opposing activities of histone acetyltransferases (HATs) and histone deaceytltransferases (HDACs). HDACs catalyze the removal of acetyl groups on the NH2-terminal lysine residues of core nucleosomal histones, and this activity is generally associated with transcriptional repression. The aberrant recruitment of HDAC activity has been associated with the development of certain human cancers (3). HDACIs are structurally varied, but share the capacity to enhance cell differentiation, induce apoptosis (4) and inhibit cancer cell growth (5). Valproic acid (VPA), a potent anticonvulsant that also acts as an HDACI, produces a paucity of side-effects in humans, even when serum levels exceed the normal therapeutic range while receiving anti-epileptic therapy (6). The drug alters the expression of a critical subset of target genes (7), and this selective modulation probably explains the therapeutic efficiency and mild adverse effects. Furthermore, VPA also has useful pharmacokinetic properties, with a significantly longer biological half-life than the other HDACIs (8). VPA has already been proposed for redifferentiating the treatment of hematological malignancies, neuroblastoma (9) and prostate cancer (10).

While VPA shows promise as a single agent for numerous tumor cells, the use of VPA in combination with other anticancer agents may be the most useful application in treating bladder cancer. The present study aimed to define the therapeutic effects of VPA in treating bladder cancer and investigated whether VPA was able to mediate the inhibition of cell growth and the induction of apoptosis in bladder cancer cells. Furthermore, the synergistic effects of VPA were examined in combination with mitomycin C (MMC), cisplatin (DDP) and adriamycin (ADM).

## Materials and methods

### Cell lines and chemicals

T24, BIU87 and 5637 bladder TCC cells were maintained in RPMI-1640 (Gibco, Carlsbad, CA, USA) supplemented with 10% fetal bovine serum (FBS). VPA and 3-(4,5-dimethylthiazol-2-yl)-2,5-diphenyltetrazolium bromide (MTT) were purchased from Sigma Chemical Co. (St. Louis, MO, USA).

### Cell viability assay

The bladder cancer cells were plated in 96-well plates at 3×10^3^ cells/well in RPMI-1640 culture medium with 10% FBS. Following a 24-h culture period, the cells were treated with medium alone or with medium containing various doses of VPA (0.5, 1, 1.5 or 3 mM) for up to 10 days. At days 1, 4, 7 and 10, the proliferation of the cells was determined using an MTT assay. Briefly, 10 μl 12 mM MTT was added to each well. Following a further 4-h incubation period, 150 μl dimethyl sulfoxide (DMSO) was added to each well and the absorbance was measured at 490 nm using a plate reader (Bio Elisa Reader ELX800; Biokit, Inc., San Diego, CA, USA). Six replicates were performed to determine each data point. The cells for the synergy effect assay were treated with medium alone or with medium containing various doses of VPA and/or chemical agents (5 mg/l DDP, 5 mg/l MMC or 2 mg/l ADM). The MTT assay was performed following 24, 48 and 72 h of treatment. The coefficient of drug interaction (CDI) was used to analyze the synergistically inhibitory effect of the drug combinations (11). The CDI was calculated as follows: CDI = AB / (A × B). According to the absorbance of each group, AB was the ratio of the combination groups to the control groups and A or B was the ratio of the single agent groups to the control group. Thus, a CDI value of <1, equal to 1 or >1 indicated that the drugs were synergistic, additive or antagonistic, respectively. A CDI of <0.7 indicated that the drugs were significantly synergistic.

### Cell morphology observation using Hoechst 33258 staining

Hoechst staining was used to visualize the apoptotic cells in the bladder cancer cell lines and cancerous tissues from the N-methyl-N-nitrosourea (MNU)-induced bladder cancer rats. The bladder cancer cells were separately incubated with 1 mM VPA and/or 5 mM DDP for 72 h. The culture medium was discarded and washed three times in ice-cold PBS. The cells were fixed with 4% paraformaldehyde and stained with 1 μg/ml Hoechst 33258 for 30 min at room temperature. The rats with MNU-induced bladder cancer were treated once a week with 25 mg/kg VPA intravesical instillation and/or 2 mg/kg DDP by intraperitoneal injection once a week for 15 weeks. The sections from the cancerous tissues were fixed with 4% paraformaldehyde and stained with 1 μg/ml Hoechst 33258 for 30 min at room temperature. The cells and tissues were observed under fluorescence microscope.

### Detecting survivin and acetylated histone H3 expression using western blotting

The T24 cells were incubated with 1 mM or 1.5 mM VPA for 72 h, then homogenized and re-suspended in Mammalian Protein Extraction reagent (M-PER; Pierce, Rockford, IL, USA). A BCA protein assay kit (Bio-Rad, Hercules, CA, USA) was used to determine the total protein concentration. The proteins were separated on a 12% Tris-HCL polyacrylamide gel (Bio-Rad) and transferred to a PVDF membrane. The membrane was blocked for an hour in blocking buffer [100 mM Tris-HCL (pH 7.5), 150 mM NaCl and 0.1% Tween-20] with 5% skimmed dry milk and incubated overnight with a 1:1,000 dilution of rabbit anti-survivin, rabbit anti-acetylated histone H3 and rabbit anti-β-actin antibodies (Cell Signaling Technology, Danvers, MA, USA), respectively, followed by anti-rabbit IgG peroxidase conjugate (1:20,000; Beyotime, Haimen, China) for 1.5 h at room temperature. The immunoreactive bands were detected using the BeyoECL Plus Western Blotting Detection System (Beyotime), according to the manufacturer's instructions.

### Flow cytometry of apoptosis by annexin V and propidium iodide (PI) double staining

The T24 cells were treated with 1 mM VPA and/or 5 mg/l DDP for 72 h. At the end of the treatment, the cells were harvested by trypsin solution to produce a single cell suspension. An annexin V and PI double staining kit (Roche Applied Science, Mannheim, Germany) was used to assess apoptosis. The cells were analyzed using flow cytometry.

### Animal studies of the effects of VPA in combination with DDP on MNU-induced bladder cancer

A cohort of 60, six to eight-week-old, female Wistar rats (Lanzhou University Experiment Animal Center, Lanzhou, Gansu, China) was used for this study, in full accordance with the National Research Council's Guide for the Care and Use of Laboratory Animals. This study was approved by the ethics committee of the School of Basic Medical Sciences, Lanzhou University (Lanzhou, China). The animals were anesthetized using intraperitoneal chloral hydrate and intravesically administered 0.15 ml 10 mg/ml MNU via a 22-gauge Teflon angiocatheter (Becton Dickinson, Sandy, UT, USA) every other week (week 1, 3, 5, 7 and 9) for a total of 5 doses following the draining of the bladder. The animals remained anesthetized for ~2 h following catheterization. Six rats were excluded from the experiment, as two died and four contracted urosepsis secondary to urethral stricture or urinary obstruction. The remaining 54 rats were divided into the following four treatment groups: 1, control (n=14); 2, VPA (n=13); 3, DDP (n=13); and 4, VPA combined with DDP (n=14). From week 11, for 15 weeks the rats were treated intravesically with 0.4 ml of either saline (groups 1 and 3) or 25 mg/kg VPA in a 0.4-ml solution (groups 2 and 4) at every first day of the week. At 2 h post-intravesical injection, the rats were treated by intraperitoneal injection with either saline (groups 1 and 3) or 2 mg/kg/dose DDP (groups 2 and 4). The animals were sacrificed at 25 weeks and a necropsy was performed. The urinary bladders were excised and the bladders were bivalved at the dome and fixed in 10% phosphate-buffered formalin for 24 h and embedded in paraffin for histopathology. The sections were stained with hematoxylin and eosin (HE). The tumors were categorized according to the histological grade using the conventional criteria. The incidence of tumor growth was scored while blinded to the treatment procedure. All the sections from each bladder specimen were reviewed under light microscopy. The section that appeared to have the greatest amount of change from the normal rat bladder was selected for histological grading. The sections were assessed and categorized into three stages: i) Hyperplasia (flat or papillary atypia or mild and moderate dysplasia); ii) superficial TCC, including Stages Pa (papillary exophytic tumors with fibrovascular cores and nuclear pleomorphism of the epithelial cells with no evidence of invasion), Pis (tumors confined to the mucosa with full mucosal thickness of marked atypia/dysplasia, including carcinoma *in situ*) and P_1_ (tumors that demonstrate evidence of lamina propria invasion); or iii) bladder wall muscle-invasive TCCs.

### Statistical analysis

The data are expressed throughout as the mean ± SEM and were analyzed using SPSS 11.0 (SPSS, Inc., Chicago, IL, USA). The data of the animal study was analyzed using the Kruskal-Wallis H test. The Wilcoxon W test was used as a post-hoc test. P<0.05 was considered to indicate a statistically significant difference.

## Results

### VPA inhibits bladder cancer cell proliferation

**Figure 1:**
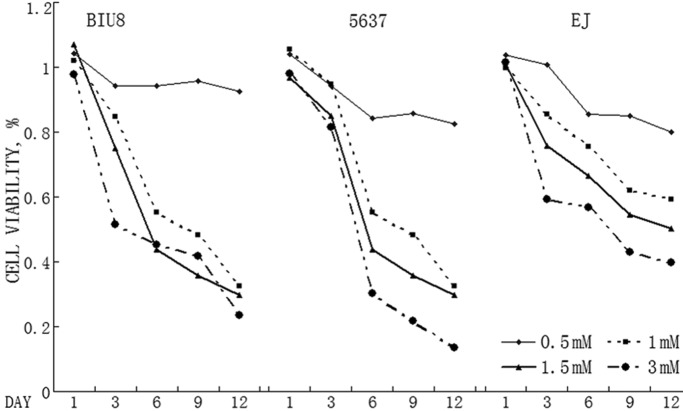
Effect of valproic acid (VPA) on bladder cancer cell survival. Cells were seeded at 3×10^3^ cells/well in 96 multiwell plates and treated with medium alone or with medium containing various doses of VPA (0.5, 1, 1.5 or 3 mM) for up to 10 days. At days 1, 4, 7 and 10, the viable cells were determined using a 3-(4,5-dimethylthiazol-2-yl)-2,5-diphenyltetrazolium bromide (MTT) assay. The VPA significantly reduced the number of surviving bladder cancer cells. The cell viability decreased in a dose-dependent manner in all the cell lines. VPA (1 mM) was able to inhibit the growth of all the bladder cancer cell lines significantly.

A panel of T24, BIU87 and 5637 bladder cancer cells were treated with various doses of VPA for up to a maximum of 10 days. VPA significantly reduced the number of viable cells in the bladder cancer cell lines (Fig. 1). The effect appeared in all three cell lines following 10 days of treatment. The cell viability decreased in a dose-dependent manner in all three cell lines. VPA (1 mM) was able to inhibit the growth of all three bladder cancer cell lines significantly. The expected plasma level for future use is 1 mM, which is clinically achievable in patients.

### VPA and/or DDP induces apoptosis in bladder cancer cell lines and in MNU-induced bladder cancer tissues

**Figure 2:**
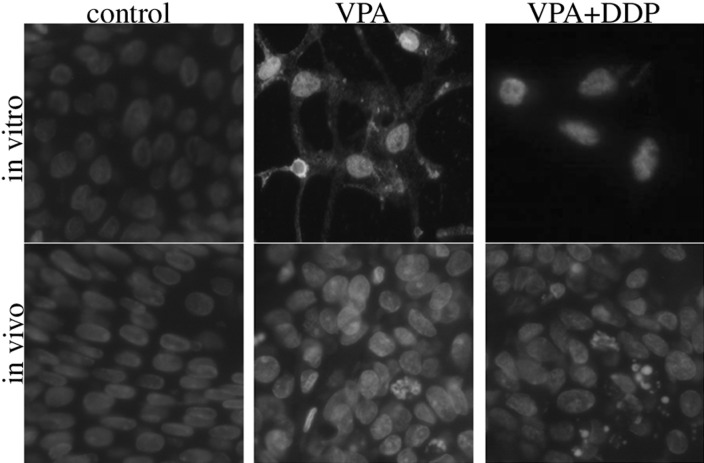
Cell morphology observations following Hoechst 33258 staining. The bladder cancer cells were separately incubated with 1 mM valproic acid (VPA) and/or cisplatin (DDP) for 72 h. The rats with bladder cancer were treated with VPA and/or DDP. The cells and sections were fixed with 4% paraformaldehyde and stained with Hoechst 33258. The group treated with VPA or VPA combined with DDP exhibited numerous apoptotic cells, while apoptosis of the control cells was not observed. Magnification, ×400.

Hoechst 33258 staining was used to determine whether apoptosis occurred following the VPA treatment. The morphology of the apoptotic bladder cancer cells *in vitro* and *in vivo* was assessed using Hoechst 33258 staining and this revealed that the viable cells displayed diffuse fluorescence in the cellular nuclei. The apoptotic cells demonstrated concentrated dense granular fluorescence. Numerous apoptotic cells were observed in the group that was treated with VPA or VPA combined with DDP. However, apoptosis of the control cells was not observed (Fig. 2).

### Synergistic effect of VPA in combination with DDP, MMC and ADM on bladder cancer cell survival

**Figure 3:**
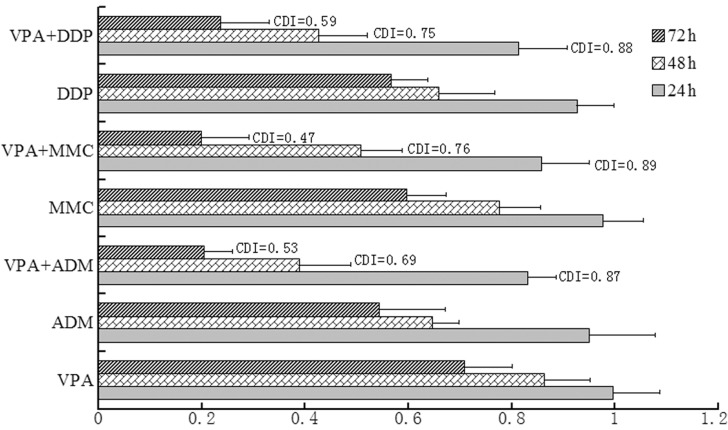
Effect of valproic acid (VPA) in combination with cisplatin (DDP), mitomycin C (MMC) and adriamycin (ADM) on bladder cancer cell survival. Using a 3-(4,5-dimethylthiazol-2-yl)-2,5-diphenyltetrazolium bromide (MTT) assay, the synergistic effects were observed in the inhibition of bladder cancer survival using a combination of 1 mM VPA with 5 mg/l DDP, 5 mg/l MMC and 2 mg/l ADM.

**Figure 4:**
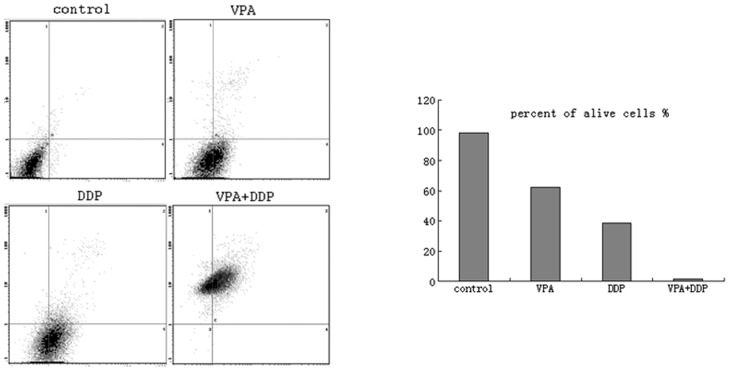
Flow cytometry of apoptosis by Annexin V and propidium iodude (PI) double staining. T24 cells were treated with valproic acid (VPA) and/or cisplatin (DDP) for 72 h. Following the treatment, the cells were harvested and apoptosis was assesed using Annexin V/PI double staining. Following the treatment with VPA, DDP or VPA and DDP, the number of apoptotic and necrotic cells increased dramatically. The number of living cells decreased significantly.

The MTT assay revealed the synergistic inhibition on the survival of bladder cancer cells by VPA combined with DDP, MMC and ADM (Fig. 3). The individual effects of VPA or DDP, MMC and ADM caused a decrease in cancer cell survival. However, when VPA was combined with DDP, MMC or ADM, the survival markedly decreased. To further confirm the growth inhibition and apoptosis induction effects of VPA on the bladder cancer cells, Annexin V and PI double staining was performed and detected using flow cytometry following VPA and/or DDP treatment for 72 h. The lower left quadrants of each of the panels in Fig. 4 show the live cells, the upper right quadrants represent the terminal apoptotic cells and the lower right quadrants represent the early apoptotic cells. Following VPA, DDP or VPA and DDP treatment, the percentage of apoptotic cells increased and the percentage of living cells decreased significantly (Fig. 4).

### VPA represses survivin and increases acetylated histone H3 expression in T24 cells

**Figure 5:**
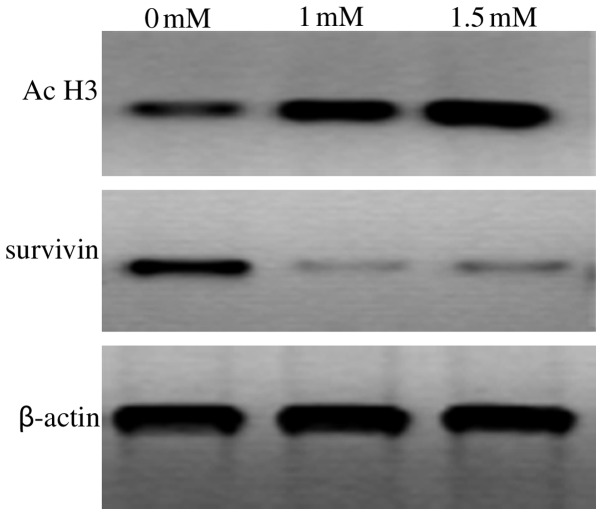
Western blot analysis of acetylated histone H3 and survivin expression in T24 cells. The T24 cells were treated with 0, 1 and 1.5 mM valproic acid (VPA) and analyzed for acetylated histone H3 and survivin expression using western blot analysis. The relative fold increase was determined by scanning densitometry of the western blot analysis normalized to β-actin. VPA treatment resulted in an increase in acetylated H3 expression and a decrease in survivin expression in the T24 cells.

The T24 cells were treated with medium alone or with medium contain VPA for 72 h, and survivin and acetylated histone H3 protein expression was detected using western blotting. As shown in Fig. 5, VPA inhibited survivin expression in the T24 cells. Acetylated histone H3 expression was increased significantly in the T24 cells following treatment with VPA at concentrations of 1 or 1.5 mM. The protein expression level in the BIU87 and 5637 cells was similar (data not shown).

### VPA and/or DDP inhibits tumor progression in MNU-induced bladder cancer

**Figure 6:**
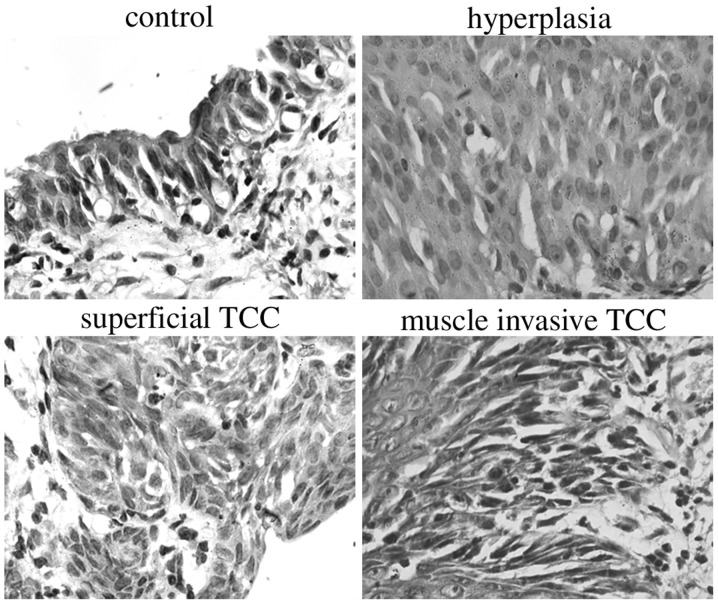
Histopathological findings in female Wistar rat bladders treated with five doses of intravesical N-methyl-N-nitrosourea (MNU). The rats commenced therapy at 11 weeks with valproic acid (VPA) and/or cisplatin (DDP) and were sacrificed at 25 weeks. The female Wistar rat bladders that were treated with five doses of MNU developed progressive neoplastic changes. These lesions progressed from hyperplasia or superficial transitional cell carcinoma (TCC) to large bulky muscle-invasive TCCs. Hematoxylin and eosin staining; magnification, ×400.

**Figure 7:**
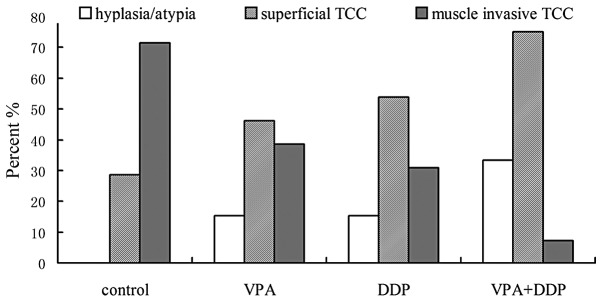
*In vivo* effects of valproic acid (VPA) and/or cisplatin (DDP) on N-methyl-N-nitrosourea (MNU)-induced bladder cancer. Histopathological findings in the female Wistar rat bladders that were treated with four doses of intravesical MNU. The rats commenced therapy at 11 weeks and were sacrificed at 25 weeks. TTC, transitional cell carcinoma.

The bladders of all the rats that were treated with intravesical MNU developed progressive neoplastic changes, which progressed into hyperplasia, superficial TCCs or bladder wall muscle-invasive TCCs (Fig. 6). The effects of VPA and/or DDP on MNU-induced bladder cancer was shown in Fig. 7. Intravesical VPA was able to prevent the progression of bladder cancer (P<0.05). Improved results were achieved using VPA combined with DDP in treating bladder cancer (P<0.01).

## Discussion

HDACIs have been shown to have an antiproliferative effect on cancer cells *in vitro*, but a number of limitations restrict their clinical use. Sodium butyrate demonstrates antitumor activity and is able to induce the differentiation of certain cancer cell lines, however, its clinical utility has been restricted by its short half-life (5 min), limiting the ability to achieve a therapeutic plasma level. Trichostatin A is also of limited therapeutic use due to its toxic side-effects *in vivo*. VPA is relatively safe, with a low toxicity *in vivo,* and has been used in the treatment of epilepsy for >30 years. Furthermore, VPA has convenient pharmacokinetic properties with a significantly longer biological half-life compared with the other HDACIs. The present study revealed that VPA at 0.5–3 mM inhibited cell proliferation and induced apoptosis in bladder cancer cells *in vitro* and *in vivo*. This range of VPA concentrations may be achieved in the serum levels of a patient when a daily dose of 20–30 mg/kg is administered for epilepsy. The VPA levels that are reached in patients who are treated for epilepsy are usually <100 mg/ml (0.7 mM). Only limited toxicity occurs when the concentration is <3.1 mM and severe side-effects develop when the concentration is >5.9 mM (12). In the present study, 1 mM VPA was able to inhibit cell proliferation and induce apoptosis dramatically (Fig. 1). Thus, 1 mM VPA is the expected plasma level for use in treating bladder cancer, as it is just above the therapeutic levels for epilepsy and appears to be clinically achievable. These data show that VPA may become a useful adjuvant therapy for cancers.

Treatment with HDACIs results in the induction of a large number of candidate genes and the repression of anti-apoptosis genes (13). In the present study, VPA was able to increase acetylated histone H3 expression in the T24 cells (Fig. 5). This indicated that VPA acts as an HDACI. The present study also revealed that VPA was able to downregulate survivin expression and induce apoptosis in bladder cancer. Survivin is a member of the inhibitors of apoptosis protein family and is involved in the inhibition of apoptosis and the regulation of cell division. Although rarely expressed in terminally differentiated normal adult tissues, survivin is upregulated in the majority of malignancies (14). The present study demonstrated that treatment with VPA dramatically and significantly increased the number of apoptotic cells and decreased survivin expression in the bladder cancer cells. It is likely that VPA induced bladder cancer cell apoptosis by downregulating survivin expression.

Histone acetylation is a significant epigenetic modification and plays a vital role in the regulation of gene expression. The process is used in combination with other anticancer agents to increase efficiency (15). Certain studies have focused on the synergistic effects of VPA (16–18). The synergistic effects of VPA have previously been considered to have no preconceived mechanistic basis. The present study revealed that the treatment with VPA results in the downregulation of anti-apoptotic proteins, including survivin. In those scenarios, VPA acts to sensitize cancer cells to various apoptotic stimuli, including chemotherapeutic drugs.

In summary, in the present study, VPA exhibited antiproliferative activity and potently induced apoptosis in the human bladder cancer cells without apparent toxic side-effects. Furthermore, VPA was able to downregulate survivin expression and increase the sensitivity of bladder cancer to chemotherapeutic dugs. The intravesical application of VPA and VPA combined with DDP was able to prevent tumor progression in rats with MNU-induced bladder cancer. These findings raise the possibility that VPA may prove particularly effective in treating bladder cancers when combined with chemotherapeutic drugs.
